# Risk Factors for Asymptomatic Enteric Pathogen Detection Among Men Who Have Sex With Men

**DOI:** 10.1093/ofid/ofz326

**Published:** 2019-07-11

**Authors:** Deborah A Williamson, Eric P F Chow, Darren Lee, Kate Maddaford, Michelle Sait, Marion Easton, Danielle Ingle, Rebecca Wigan, Vesna De Petra, Benjamin P Howden, Christopher K Fairley, Marcus Y Chen

**Affiliations:** 1 Microbiological Diagnostic Unit Public Health Laboratory, Department of Microbiology and Immunology, The University of Melbourne at The Peter Doherty Institute for Infection and Immunity, Australia; 2 Melbourne Sexual Health Centre, Alfred Health, Carlton, Victoria, Australia; 3 Central Clinical School, Monash University, Melbourne, Australia; 4 Victorian Department of Health and Human Services, Melbourne, Australia

**Keywords:** enteric pathogens, epidemiology, gastrointestinal disease, sexually transmitted infections

## Abstract

Improved knowledge of factors that promote outbreaks of enteric pathogens among men who have sex with men (MSM) could enable targeted public health interventions. We detected enteric pathogens in 57 of 519 (11%) asymptomatic MSM, and we found that enteric pathogen detection was associated with both oroanal sex (rimming) and group sex.

Gastrointestinal infections are a major global cause of morbidity. Transmission of enteric pathogens occurs predominantly by the fecal-oral route, through ingestion of contaminated food or water. Sexual transmission is also a well recognized mode of transmission, mainly amongst men who have sex with men (MSM), and, over the past decade, there have been increasing reports of outbreaks of enteric pathogens among MSM, including *Shigella* spp, Shiga-toxin-producing *Escherichia coli* (STEC), and hepatitis A virus [1–7]. In particular, sexually transmitted shigellosis has emerged as a major public health concern, mainly due to high rates of resistance to clinically relevant antimicrobials [4, 5, 8]. In our setting, we recently described the cocirculation of 2 major drug-resistant *Shigella* lineages among MSM over a 27-month period, with each lineage including at least 50 men [5]. To date, however, the biological and behavioral factors that drive the persistence of shigellosis epidemics in MSM are not well defined.

Given the extent and persistence of *Shigella* in MSM in our setting, we hypothesised that asymptomatic carriage of enteric pathogens, including *Shigella*, could act as a potential reservoir for gastrointestinal outbreaks in MSM, and that improved knowledge of factors that promote outbreaks of enteric disease could enable public health interventions to be directed towards those at highest risk of disease. To address these hypotheses, we performed a cross-sectional study of asymptomatic MSM in Melbourne, Australia, and we screened for the presence of 15 bacterial, viral, and protozoan enteric pathogens. Furthermore, we examined the association between behavioral risk factors and the presence of asymptomatic enteric pathogens to better understand the drivers of sexually transmitted enteric infections in MSM.

## METHODS

### Setting, Patients, and Data Sources

Melbourne Sexual Health Centre is the major publicly funded sexual health center in Victoria, Australia, with approximately 50 000 consultations annually [9]. The center consists of a free walk-in sexually transmitted infections (STI) clinic and outpatient human immunodeficiency virus (HIV) and HIV pre-exposure prophylaxis (PrEP) clinics. Between November 1, 2018 and February 28, 2019, we undertook a cross-sectional study to estimate the prevalence of enteric pathogens in asymptomatic MSM (those who did not have diarrhea in the 2 weeks before testing) attending for STI screening. Recruitment focused on 3 groups of MSM: HIV-negative men currently taking PrEP, HIV-negative men not taking PrEP, and HIV-positive men. All men completed a brief questionnaire (Supplementary Appendix) that captured information on age, HIV status, use of PrEP, and diarrhea (defined as ≥3 loose or liquid stools within the last 2 weeks). Men were asked about sexual practices, including group sex in the last month, use of party drugs in the last month, and insertive rimming (mouth or tongue touching another man’s anus) within the last 12 months.

### Microbiological Testing

Routine STI screening consisted of an oropharyngeal swab, first pass urine, and an anal swab for *Neisseria gonorrhoeae* and *Chlamydia trachomatis* nucleic acid amplification testing using the Aptima Combo 2 assay (Hologic). Men who agreed to the study had another anal swab collected using an ESwab (Copan Diagnostics Inc., Brescia, Italy). Deoxyribonucleic acid (DNA) was extracted using the QIASymphony DSP Virus/Pathogen Midi Kit (QIAGEN) protocol according to the manufacturer’s instructions. Extracted DNA was tested on a High-Plex24 system using the Faecal Pathogen M 16-well assay (AusDiagnostics Pty. Ltd., Sydney, Australia), a multiplexed tandem polymerase chain reaction (PCR) assay that can detect 15 enteric pathogens (Supplementary Table 1).

### Statistical Analysis

Proportions of enteric pathogens were calculated with 95% binomial confidence intervals (CIs). Associations with enteric pathogen detection were assessed using univariate and multivariable logistic regression analyses. Sexual behaviors with *P* < .10 in the univariable analyses were included in the multivariable model. Statistical analyses were performed in STATA (version 14.2; Stata Corporation, College Station, TX).

### Ethics

Ethical approval was obtained from the Alfred Hospital Ethics Committee (Project 271/18).

## RESULTS

### Characteristics of Study Population and Prevalence of Enteric Pathogens

Overall, 519 MSM were included in the study, with ages ranging between 18 and 73 years (median 31 years; interquartile range 26–40 years). A total of 78 of 519 men (15.0%; 95% CI, 12.2%–18.3%) were HIV-positive, and of the remaining HIV-negative men, 227 of 441 (51.5%; 95% CI, 46.8%–56.1%) were taking PrEP. Overall, 363 of 505 MSM (71.9%; 95% CI, 67.8%–75.6%) reported insertive rimming in the last 12 months, 123 of 519 (23.7%; 95% CI, 20.2%–27.6%) reported group sex in the last month (with the number of episodes ranging from 1 to 30), and 68 of 519 (13.1%; 95% CI, 10.5%–16.3%) men reported using party drugs in the last month (with the number of episodes ranging from 1 to 20).

### Prevalence and Risk Factors for Enteric Pathogen Detection

Rectal swabs from 57 of 519 (11.0%; 95% CI, 8.4%–14.0%) men tested positive for any enteric pathogen (Supplementary Table 2). The most common pathogen detected was *Campylobacter* spp (13 of 519; 2.5%; 95% CI, 1.5%–4.3%), followed by astrovirus (10 of 519; 1.9%; 95% CI, 1.0%–3.6%), *Yersinia* spp (9 of 519; 1.7%; 95% CI, 0.8%–3.3%), and Shiga-toxin-producing *E coli* (9 of 519; 1.7%; 95% CI, 0.8%–3.3%) (Supplementary Table 2). *Shigella* spp were detected in 5 of 519 men (1.0%; 95% CI, 0.3%–2.3%). Two enteric pathogens were detected in 8 of 519 men (1.5%; 95% CI, 0.7%–3.1%) (Supplementary Table 2).

There was no difference in the prevalence of enteric pathogen carriage between different age groups or HIV status (Table 1). Likewise, there was no significant difference in enteric pathogen carriage between PrEP and non-PrEP users (Table 1). However, when behavioral factors were adjusted, the prevalence of enteric pathogen detection was independently associated (1) with men who reported insertive rimming (adjusted odds ratio [aOR] = 3.32; 95% CI, 1.38–7.97) and (2) with men who reported group sex (aOR = 2.00; 95% CI, 1.11–3.60) (Table 1).

**Table 1.** Associations Between Men Who Have Sex With Men and Enteric Pathogen Detection

Characteristics	Detection of an Enteric Pathogen, n/N (%)	OR (95% CI)	*P* Value	Adjusted OR (95% CI)	*P* Value
Age (Years)					
18–24	9/84 (10.7%)	1 (ref)			
25–34	29/240 (12.1%)	1.15 (0.52–2.53)	.737		
≥35	19/195 (9.7%)	0.90 (0.39–2.08)	.805		
HIV and PrEP Status					
HIV negative, no PrEP	20/214 (9.3%)	1 (ref)			
HIV negative, on PrEP	29/227 (12.8%)	1.42 (0.78–2.60)	.254		
HIV positive	8/78 (10.3%)	1.11 (0.47–2.63)	.815		
Rimming in the Last 12 Months					
No	6/142 (4.2%)	1 (ref)		1 (ref)	
Yes	51/363 (14.0%)	3.71 (1.55–8.84)	.003	3.32 (1.38–7.97)	.007
Can’t remember	0/14 (0%)	-	-	-	-
Group Sex in the Last Month					
No	35/396 (8.8%)	1 (ref)		1 (ref)	
Yes	22/123 (17.9%)	2.25 (1.26–4.00)	.006	2.00 (1.11–3.60)	.021
Used Party Drugs in the Last Month					
No	47/451 (10.4%)	1 (ref)			
Yes	10/68 (14.7%)	1.48 (0.71–3.09)	.295		
Sex Overseas in the Last 12 Months					
No	15/165 (9.1%)	1 (ref)			
Yes	16/105 (15.2%)	1.80 (0.85–3.81)	.126		
Unknown/Declined/Missing	26/249 (10.4%)	1.17 (0.60–2.27)	.653		
Pharyngeal Gonorrhea					
Negative	50/460 (10.9%)	1 (ref)			
Positive	3/29 (11.1%)	0.95 (0.28–3.24)	.930		
Not tested	4/30 (12.1%)	1.26 (0.42–3.76)	.677		
Urethral Gonorrhea					
Negative	51/486 (10.5%)	1 (ref)			
Positive	1/6 (16.7%)	1.71 (0.20–14.89)	.629		
Not tested	5/27 (18.5%)	1.94 (0.70–5.34)	.200		
Anorectal Gonorrhea					
Negative	51/468 (10.9%)	1 (ref)			
Positive	2/18 (11.1%)	1.02 (0.23–4.57)	.977		
Not tested	4/33 (12.1%)	1.13 (0.38–3.34)	.828		
Pharyngeal Chlamydia					
Negative	51/486 (10.5%)	1 (ref)			
Positive	2/7 (28.6%)	3.41 (0.65–18.04)	.149		
Not tested	4/26 (15.4%)	1.55 (0.51–4.68)	.436		
Urethral Chlamydia					
Negative	52/475 (10.9%)	1 (ref)			
Positive	0/17 (0%)	-			
Not tested	5/27 (18.5%)	1.85 (0.67–5.09)	.234		
Anorectal Chlamydia					
Negative	44/442 (10.0%)	1 (ref)			
Positive	9/44 (20.5%)	2.33 (1.05–5.16)	.038		
Not tested	4/33 (12.1%)	1.25 (0.42–3.71)	.691		

Abbreviations: CI, confidence interval; HIV, human immunodeficiency virus; OR, odds ratio; PrEP, pre-exposure prophylaxis.

Overall, a total of 85 of 519 (16.4%) individuals had concurrent gonorrhea (oropharyngeal [29 of 489; 5.9%], anorectal [18 of 486; 3.7%], urethral [6 of 492; 1.2%]) or chlamydia (oropharyngeal [7 of 493; 1.4%], anorectal [44 of 486; 9.1%], urethral [17 of 492; 3.5%]). Of note, men with anorectal chlamydia had 2.33 (95% CI, 1.025–5.16) times more odds of having an enteric pathogen detected (Table 1).

## DISCUSSION

In this study, 11% of asymptomatic MSM had at least 1 viral or bacterial enteric pathogen detected, with detection associated with both insertive rimming and group sex. To our knowledge, our study represents the first to link asymptomatic enteric pathogen carriage in MSM with specific behavioral risk factors. Although sexual transmission of enteric pathogens has been recognized for several decades, as early as the 1970s when the term “gay bowel syndrome” was used [10], there remains limited understanding of the specific drivers for outbreaks of enteric infections in MSM, including the role of asymptomatic carriage in facilitating transmission.

Over a 4-month period, we detected a range of enteric pathogens, including pathogens associated with outbreaks among MSM. For example, *Campylobacter* spp, identified in 2.5% of asymptomatic MSM in our study, has previously been associated with gastrointestinal outbreaks in MSM, including an outbreak of erythromycin- and ciprofloxacin-resistant *Campylobacter jejuni* in Quebec, Canada [11], and it has previously been identified in 1.8% of MSM with confirmed rectal chlamydia [12]. Likewise, STEC, identified in 1.7% of MSM in this study, was described in an MSM-associated outbreak in the United Kingdom (UK) in 2014 [3]. Of note, genomic analysis of isolates from this UK outbreak demonstrated the presence of shared antimicrobial resistance determinants (most notably the *mph*(A) gene encoding azithromycin resistance) between STEC and *Shigella* spp [4]. This finding is of particular relevance in our setting, where we recently identified azithromycin resistance rates of over 90% in MSM-associated *Shigella* [5]. Although we only detected *Shigella* spp in 5 individuals (1.0%), the relatively high number of partners some MSM have places them at significant risk, even with this low prevalence. For example, the Australian National Pleasure and Sexual Health (PASH) study found that, among 1590 MSM who reported having sex with casual partners in the preceding 6 months, the median number of partners was 6, with at least 60% of the 1590 MSM engaging in rimming [13]. It is likely that frequent oroanal contact, coupled with asymptomatic carriage of a highly infectious pathogen, and fueled by selection pressure from azithromycin, are drivers of shigellosis in MSM in our setting.

Previous work has shown that direct oroanal contact (rimming) is associated with acute, symptomatic enteric infections, including shigellosis and STEC [14, 15]. Our observation that enteric pathogen carriage is independently associated with group sex supports the potential for not only direct oroanal acquisition, but also indirect acquisition. However, we did not collect the number of insertive rimming partners, so our finding that group sex was associated with enteric pathogen detection may be confounded by the number of partners during group sex.

Our exploratory study had a number of limitations, and it raised several important questions for future work. First, our study was conducted at a single sexual health service in Australia and may not be generalizable to all MSM. Second, we did not collect detailed epidemiological data to allow us to adjust for all possible risk factors such as occupational exposure (eg, to animals or childcare), contact with an affected individual, or individual hygiene habits. However, our observation of an association between detection of an enteric pathogen with oroanal sex is biologically plausible and has been found in symptomatic individuals with similar organisms [13, 14]. Third, we only assessed enteric pathogen detection in 1 group of individuals (ie, MSM)—future studies should incorporate both additional epidemiological risk data, and a control population (eg, asymptomatic heterosexuals) to validate the findings from our study, and identify broader (if any) risk factors. Moreover, the detection of additional pathogens not previously associated with enteric disease in MSM (*Yersinia* spp and astrovirus) should be further explored, including the potential role of these pathogens in symptomatic outbreaks of gastrointestinal disease in MSM. Finally, the high sensitivity of PCR-based detection of enteric pathogens raises interpretative challenges at a patient-level (eg, risk of developing infection and risk of transmission) that should be further explored in larger cohort studies.

## CONCLUSIONS

Regardless of these limitations, our findings have direct public health implications for the prevention of gastrointestinal infections particularly in relationship to the role of asymptomatic carriage. We found that 11% of MSM had a pathogen detected and that oroanal sex is common. In this context, it is likely that a substantial incidence of infection relates to asymptomatic transmission during oroanal sex. More important, a recent study asked MSM attending an STI clinic what sexual practices they would be likely to forego to prevent an STI, and approximately 50% said they would be willing to stop rimming [16]. Men who have sex with men should be advised in health promotional messages that the practice of rimming increases the risk for carriage of asymptomatic enteric pathogens.

## Supplementary Data

Supplementary materials are available at *Open Forum Infectious Diseases* online. Consisting of data provided by the authors to benefit the reader, the posted materials are not copyedited and are the sole responsibility of the authors, so questions or comments should be addressed to the corresponding author.

ofz326_suppl_supplementary_tables
