# Supplementary material for: Risk Factors for Asymptomatic Enteric Pathogen Detection Among Men Who Have Sex With Men
Source: Open Forum Infect Dis. 2019 Jul 11;6(9):ofz326. doi: 10.1093/ofid/ofz326 (PMC6798250; doi:10.1093/ofid/ofz326)
Supplement: ofz326_suppl_supplementary_tables [file ofz326_suppl_supplementary_tables.docx]

**Supplementary Table 1.** Enteric pathogens detected by the Faecal Pathogens M (16-well) multiplex tandem PCR panel (AusDiagnostics Pty Ltd.)

| Pathogen |
| --- |
| ***Bacteria*** |
| *Salmonella* spp. |
| *Campylobacter* spp. including *C. jejuni, C. coli, C. doyeli* |
| *Shigella* spp. |
| Shigatoxin (Stx) 1 and 2 |
| *Clostridium difficile* (Toxin B containing strains) |
| *Yersinia spp.* |
| *Aeromonas* *hydrophila* |
| ***Viruses*** |
| Norovirus genotypes G.1 **and G**.2 |
| Rotavirus |
| Astrovirus |
| Adenovirus groups F and G |
| ***Parasites*** |
| *Giardia lamblia* |
| *Entamoeba histolytica* |
| *Cryptosporidium spp.* including *C.* *hominis*, C. *parvum, C. wrairi, C. meleagridis* |

**Supplementary Table 2.** Enteric pathogens detected in 519 men who have sex with men (MSM) attending Melbourne Sexual Health Centre between 1st November 2018 and 28th February 2019.

| Pathogen(s)* | Detection of an enteric pathogen, n |
| --- | --- |
| *Campylobacter* spp. | 13 |
| *Yersinia* spp. | 9 |
| Astrovirus | 10 |
| Stx1 | 9 |
| *Giardia* spp. | 7 |
| *Shigella* spp | 5 |
| *Aeromonas* spp | 2 |
| *Entamoeba histolytica* | 2 |
| Norovirus G.1 | 2 |
| Rotavirus | 3 |
| *Salmonella* spp. | 2 |

*includes eight patients with two enteric pathogens (*Campylobacter* and Stx1; rotavirus and astrovirus; *Shigella* spp. and astrovirus; *Shigella* spp. and rotavirus; Stx1 and astrovirus; Stx1 and *Giardia*; *Yersinia* spp. and astrovirus; *Yersinia* spp. and *Giardia*).
